# Fabrication of Innovative Silk/Alginate Microcarriers for Mesenchymal Stem Cell Delivery and Tissue Regeneration

**DOI:** 10.3390/ijms18091829

**Published:** 2017-08-23

**Authors:** Sara Perteghella, Elisa Martella, Laura de Girolamo, Carlotta Perucca Orfei, Michela Pierini, Valentina Fumagalli, Domenica Valeria Pintacuda, Theodora Chlapanidas, Marco Viganò, Silvio Faragò, Maria Luisa Torre, Enrico Lucarelli

**Affiliations:** 1 Department of Drug Sciences, University of Pavia, Viale Taramelli 12, 27100 Pavia, Italy; sara.perteghella@unipv.it (S.P.); michela.pierini@ior.it (M.P.); valentina.fumagalli@unipv.it (V.F.); valeria.pintacuda@unipv.it (D.V.P.); theodora.chlapanidas@unipv.it (T.C.); 2 Department of Biomedical and Neuromotor Sciences (DIBINEM), University of Bologna, Via Zamboni 33, 40126 Bologna, Italy; elisa.martella@isof.cnr.it; 3 Osteoarticular Regeneration Laboratory, 3rd Orthopaedic and Traumatologic Clinic, Rizzoli Orthopaedic Institute, Via Giulio Cesare Pupilli 1, 40136 Bologna, Italy; enrico.lucarelli@ior.it; 4 IRCCS Istituto Ortopedico Galeazzi, Via R. Galeazzi 4, 20161 Milan, Italy; laura.degirolamo@grupposandonato.it (L.d.G.); carlotta.perucca@grupposandonato.it (C.P.O.); marco.vigano@grupposandonato.it (M.V.); 5 Silk Division, Innovhub, Stazioni Sperimentali per l’Industria, Via G. Colombo 83, 20133 Milan, Italy; silvio.farago@mi.camcom.it

**Keywords:** microcarriers, alginate, silk fibroin, mesenchymal stem cells, regenerative medicine, musculoskeletal tissues

## Abstract

The aim of this study was to exploit silk fibroin’s properties to develop innovative composite microcarriers for mesenchymal stem cell (MSCs) adhesion and proliferation. Alginate microcarriers were prepared, added to silk fibroin solution, and then treated with ethanol to induce silk conformational transition. Microcarriers were characterized for size distribution, coating stability and homogeneity. Finally, in vitro cytocompatibility and suitability as delivery systems for MSCs were investigated. Results indicated that our manufacturing process is consistent and reproducible: silk/alginate microcarriers were stable, with spherical geometry, about 400 μm in average diameter, and fibroin homogeneously coated the surface. MSCs were able to adhere rapidly onto the microcarrier surface and to cover the surface of the microcarrier within three days of culture; moreover, on this innovative 3D culture system, stem cells preserved their metabolic activity and their multi-lineage differentiation potential. In conclusion, silk/alginate microcarriers represent a suitable support for MSCs culture and expansion. Since it is able to preserve MSCs multipotency, the developed 3D system can be intended for cell delivery, for advanced therapy and regenerative medicine applications.

## 1. Introduction

In 1967, van Wezel introduced for the first time the concept of microcarriers as a method to produce large scale vaccines and to improve adherent mammalian cell growth [1]. Nowadays, in regenerative medicine applications, microcarriers can be used as a fast and reliable tool for ex vivo cell expansion, as well as a vehicle to deliver cells to a target tissue [2]. A wide range of microcarriers with different properties (degree of porosity, chemical composition and surface topography) and diameter, comprised between 100 and 400 µm, are now commercially available [3,4].

When microcarriers act as a delivery system, cells are maintained for a longer time period in the lesion site [5], giving them the possibility to secrete growth factors and actively participate in tissue matrix deposition, promoting its regeneration [2,6]. Many different natural or synthetic biopolymers have been investigated for microcarrier formulation for regenerative medicine [7,8,9,10]. Commonly employed for cell and drug/growth factor encapsulation [11,12,13,14], alginate is a bedrock biomaterial for cell transplantation [15] due to its properties of fast sol-gel transition in contact with divalent cations, in vivo biocompatibility, permeability, and dissolution [16]. However, alginate surface is unsuitable for cell adhesion due to the presence of negative charges and its deficiency of integrin domains [17,18]. To overcome this inconvenience, alginate can be conjugated with an arginine-glycine-asparagine (RGD) sequence to increase its cell adhesion properties [19] or combined with natural proteins, such as silk fibroin. Due to its peculiar characteristics, such as versatility [20,21], biodegradability and biocompatibility [22,23], silk fibroin is widely used to develop scaffolds for different applications, mainly to be used in association with cells. Indeed, silk fibroin is able to promote cell adhesion, proliferation and differentiation particularly of mesenchymal stem/stromal cells (MSCs) [24,25,26,27,28,29,30,31]. 

MSCs have been widely studied for regenerative medicine applications due to their immune-privileged nature and multi-lineage differentiation ability [32,33], and they are currently employed in more than 700 clinical trials (Available online: http://www.clinicaltrials.gov). Their use ranges from immune (e.g., graft-versus-host-disease, Crohn’s disease) to degenerative/post-traumatic pathologies. In particular in the orthopaedic field, cell-based therapies have been proposed to treat bone, cartilage, ligament, tendon and muscle injuries [7,9,34,35,36,37]. Adipose tissue is one of the most convenient sources of MSCs due to its availability and accessibility. Moreover, the yield of MSCs from adipose tissue (adipose-derived stem/stromal cells, ASCs) is higher than that of bone marrow MSCs [38,39], as well as their immunomodulatory properties [40,41]. 

Jo and colleagues [42] recently proposed a matrix constituted by alginate/hydroxyapatite/silk fibroin as scaffold for bone tissue regeneration, in order to reduce the immune reaction after in vivo implantation: no evidence of the inflammatory reaction or giant cell formation was observed around the graft material, in rats’ central calvarial bone defects.

In the present work we developed and characterized composite microcarriers constituted by a core of alginate and a silk fibroin shell, evaluating their in vitro cytocompatibility and suitability as a delivery system for ASCs in regenerative medicine applications. This approach could open new insights toward the development of an injectable microcarrier-based system aimed at the local delivery of cells to the injury site, focusing on the treatment of musculoskeletal tissue as a potential use in joint-related pathologies, such as early osteoarthritis and intervertebral disks.

## 2. Results

### 2.1. Production Process

**Figure 1:**
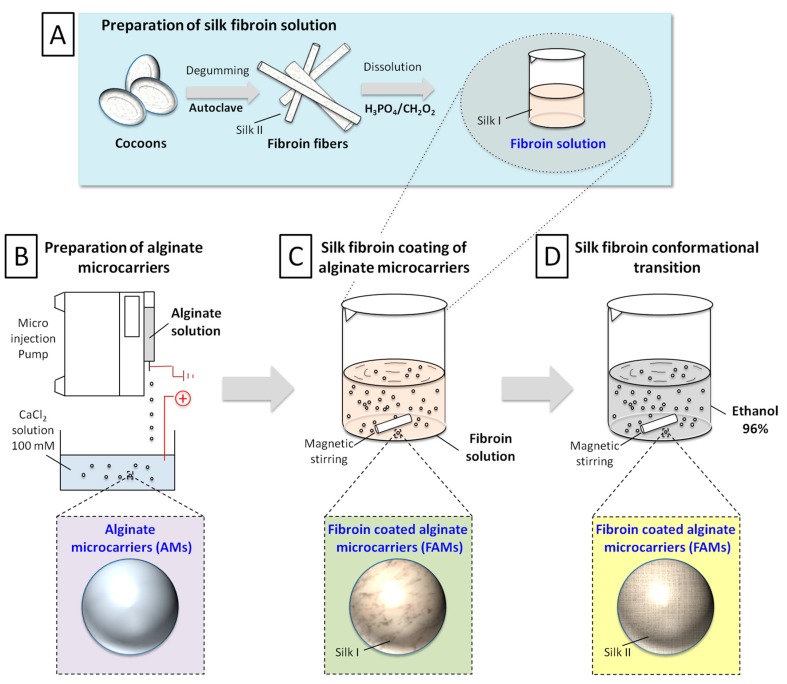
Schematic representation of production process: preparation of silk fibroin solution (**A**); Preparation of alginate cores (**B**); Coating of alginate cores with silk fibroin solution obtaining fibroin/alginate microcarriers (**C**); Silk fibroin conformational transition, obtaining stable composite devices (**D**).

The novel production process of fibroin-coated alginate microcarriers (FAMs) has been structured in four steps (Figure 1): preparation of silk fibroin solution (Figure 1A); preparation of alginate cores (alginate microcarriers, AMs, Figure 1B); coating of alginate cores with silk fibroin solution obtaining fibroin/alginate microcarriers (Figure 1C); and silk fibroin conformational transition, obtaining stable composite devices (Figure 1D).

The production process of FAMs has been developed and standardized in lab-scale, leading to a consistent technology, verified in five independent runs performed during one year. Silk fibroin dissolution, following a previously defined protocol, allowed the conformational transition from silk II (insoluble β-sheet conformation) to silk I (water-soluble structure). AMs were prepared using a bead generator after parameter optimization (alginate flow rate, nozzle diameter, voltage magnitude, calcium chloride concentration, magnetic stirring rate, distance between the needle tip and gelling bath, data not shown). Subsequently, AMs were mildly shaken into the silk fibroin solution and finally treated with ethanol to reconvert soluble fibroin (silk I) into a stable, insoluble homogeneous fibroin coating (Silk II) [43].

### 2.2. Physico-Chemical and Morphological Characterization of Microcarriers

**Figure 2:**
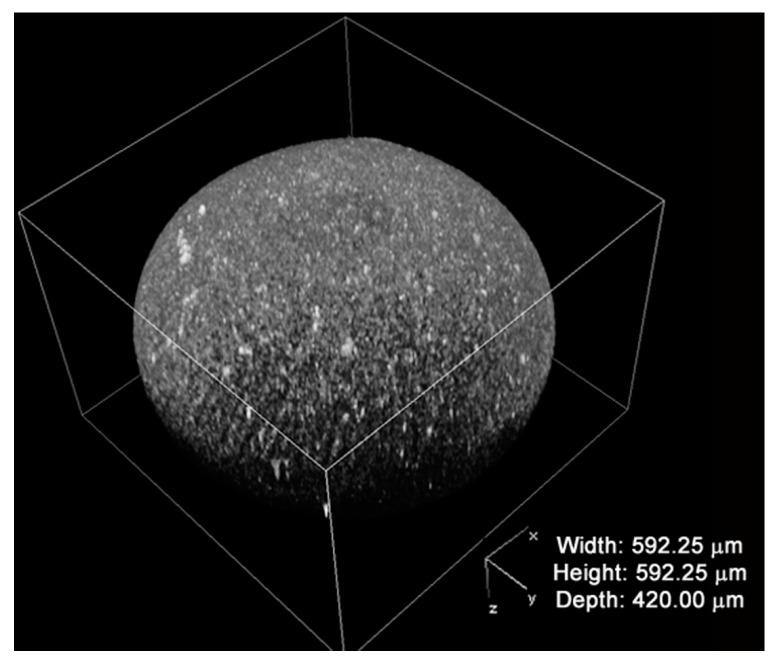
Fibroin coating reconstruction by confocal microscopy of fibroin-coated alginate microcarriers (FAMs).

The distribution of the fibroin coating has been investigated with confocal microscopy by exploiting the fluorescence emitted by the fibroin when it was excited with laser. Figure 2 shows a representative picture showing a 3D reconstruction of the fibroin coating florescence of FAMs. The fibroin completely covered the surface of the microcarrier, and numerous and scattered clots can be distinguished on the surface of the microcarrier.

**Figure 3:**
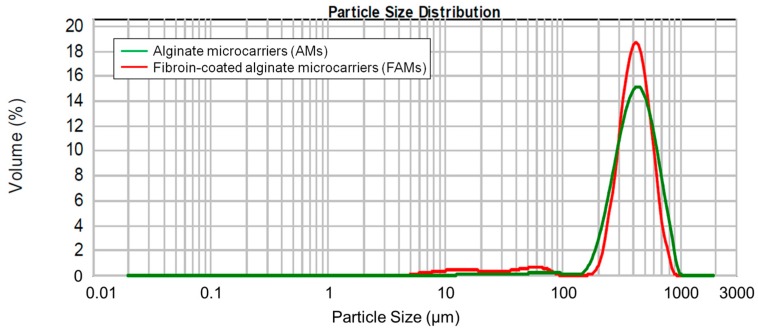
An illustrative particle size distribution of alginate microcarriers (AMs) (green line) and FAMs (red line). Data are reported as volume percentage values.

**Figure 4:**
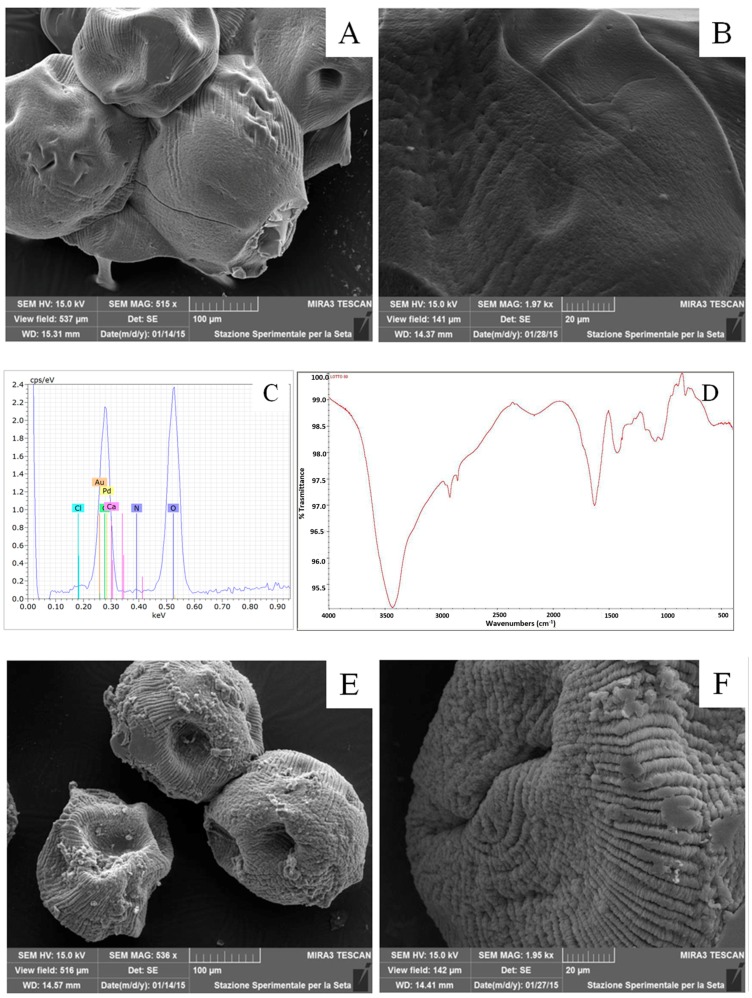

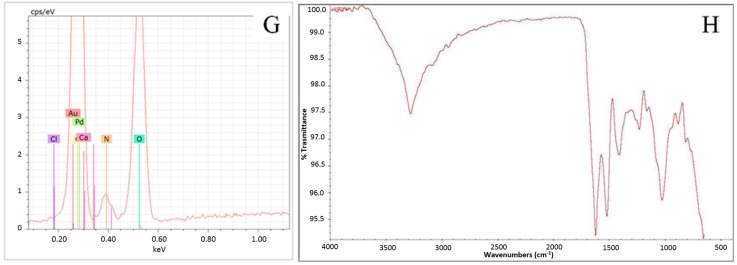
Morphological and physico-chemical characterization of AMs (**A**–**D**) and FAMs (**E**–**H**). Scanning electron microscope (SEM) images: AMs smooth surface (**A**,**B**) and FAMs rough surface (**E**,**F**); energy dispersive X-ray (EDX) spectra: fibroin nitrogen peak in FAMs spectrum (**G**), and absence of the fibroin nitrogen peak in AMs spectrum (**C**); Fourier transform infrared spectroscopy (FTIR) spectra: (**D**) AMs calcium alginate peak stretching vibrations of O–H bonds in the range 3000–3600 cm^−1^, stretching vibrations of aliphatic C–H at ~2900 cm^−1^ and asymmetric stretching vibration of the carboxylate group at ~1600 cm^−1^ (**D**); (**H**) absorption bands of FAMs fibroin crystalline β-sheets conformation at ~1620 cm^−1^ for Amide I and ~1520 cm^−1^ for Amide II.

Before and after the fibroin coating process, the microcarriers were characterized in terms of particle size distribution, morphology, and fibroin molecular conformation. Figure 3 reports the microcarrier size distribution curve. The coating process did not influence the microcarrier size distribution as evidenced by the absence of a significant difference in volume weighted mean distribution, before and after coating (*p* > 0.05). In particular, AMs showed a diameter of 464.34 ± 62.190 µm (*n* = 5) in respect to FAMs that showed a particle size of 421.94 ± 46.003 µm (*n* = 10). Statistical analysis demonstrated no significant differences between AMs and FAMs. The slight reduction of microcarrier particle size after fibroin coating might be ascribed to the production process; in fact, the treatment with ethanol could cause a slight dehydration of alginate core with a consequent slight decrease in particle size. The morphological characterization of microcarriers was performed by scanning electron microscopy (SEM): AMs appeared as spherical structures with a smooth surface (Figure 4A,B), whereas the fibroin coating formed a shell with a rough surface (Figure 4E,F). The elemental analysis of the microcarrier surface was performed by energy dispersive X-ray (EDX) analysis and a well-defined peak corresponding to nitrogen confirmed the presence of silk fibroin in FAMs (Figure 4G), with respect to AMs (Figure 4C).

The fibroin molecular conformation was evaluated analyzing the Fourier transform infrared spectroscopy (FTIR) spectra on both AMs and FAMs. The FTIR spectrum of AMs showed peculiar absorption bands of calcium alginate which is the unique polymeric component of the microsystem. Stretching vibrations of abundant O–H bonds in the range 3000–3600 cm^−1^, stretching vibrations of aliphatic C–H at ~2900 cm^−1^ and asymmetric stretching vibration of the carboxylate group at ~1600 cm^−1^ are visible in Figure 4D. 

The FTIR spectrum of FAMs (Figure 4H) confirmed the presence of silk fibroin, and in details, the conformational transition of silk fibroin after ethanol treatment as shown by the main absorption bands detected at ~1620 cm^−1^ for Amide I (C=O stretching) and ~1520 cm^−1^ for Amide II (C–N stretching and N–H bending). These peaks corresponded to the typical vibrational absorption of β-sheets protein secondary structure, indicating that fibroin existed on the microcarrier surface in its stable conformation, after ethanol treatment (Figure 4H) [30,31,44].

### 2.3. Human Adipose Derived Stem Cells (hASCs) Viability and Proliferation

**Figure 5:**
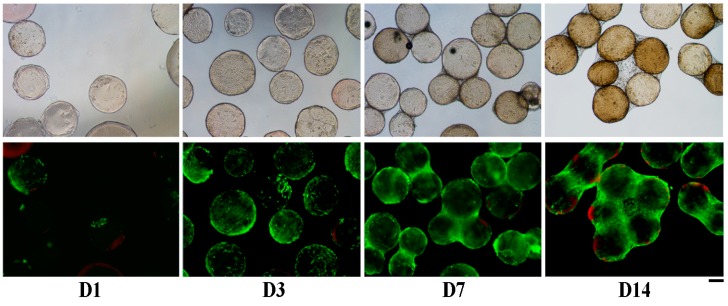
Phase contrast (top) and fluorescence microscopy (bottom) images of Live and Dead stained cells (green: live cells; red: dead cells) with FAMs at 1, 3, 7, and 14 days (D1, D3, D7, D14) from cell seeding. Scale bar = 200 µm.

**Figure 6:**
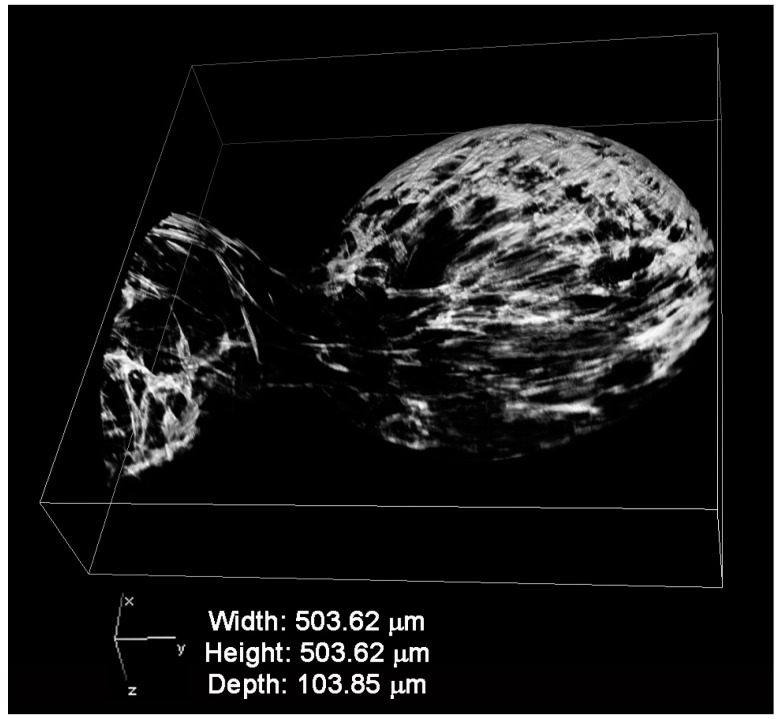
Confocal microscopy image of human adipose derived stem cells (hASCs) F-actin cytoskeleton distribution on FAM surface after 7 days from cell seeding.

In order to evaluate their cytocompatibility, FAMs were cultured with hASCs. Cells were stained with Calcein-AM and Ethidium Homodimer-1 to evaluate live (green) and dead (red) cells, respectively (Figure 5). At day one, numerous cells adhered to the surface of FAMs, even if they were not homogeneously distributed. After 3 days of culture, the cells almost completely covered the surface of the FAMs, whereas at day 7, cells started to build connections among FAMs. At 14 days from cell seeding, most of the FAMs were linked together by cells, and numerous tridimensional structures were created by interaction between adherent cells. No dead cells were detected on the FAMs surface along time in culture, thus confirming the good cytocompatibility of FAMs. As expected, after 14 days of culture the total number of adherent cells was increased and only about 10% of dead cells were detected; these results confirmed the good cytocompatibility of FAMs. The actin staining of hASCs with fluorescent phalloidin after 7 days of culture allowed researchers to observe that the cells strongly adhered to the fibroin coating, with a “stretching” shape morphology adapted to the curvature of the microcarrier surface (Figure 6).

**Figure 7:**
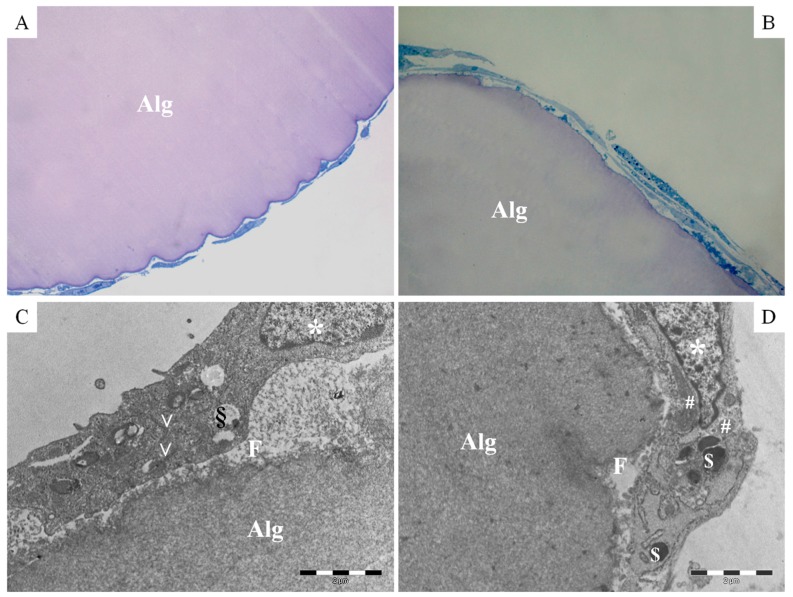
Transversal sections of hASCs cultured on FAMs after 7 (**A**,**C**) and 14 (**B**,**D**) days of culture; (**A**,**B**) light microscopy microphotographs, Toluidine Blue Staining, magnification 20×; (**C**,**D**) Transmission electron microphotographs, 2 µm of scale bar: Alg alginate; F fibroin; * nucleus; V rough endoplasmic reticulum; # mitochondria; § vacuoles; $ lysosomes.

These data were confirmed by the ultrastructural analysis of the transversal sections of FAMs, which showed a well-defined outline of alginate microcarriers and a silk fibroin shell (thickness about 0.2 µm, Figure 7). Human ASCs tightly adhered to the FAM surface and exhibited their characteristic fibroblast-like shape. Moreover, a continuous and regular cell membrane has been appreciated and typical cytoplasm components such as nucleus, rough endoplasmic reticulum, vesicles, mitochondria, vacuoles and lysosomes were observed.

**Figure 8:**
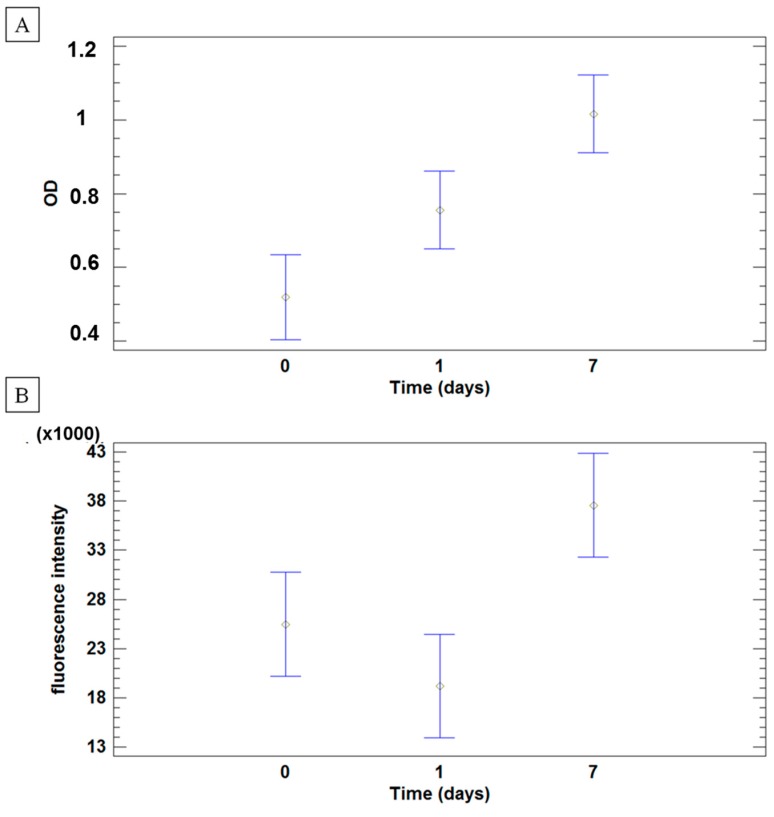
hASCs metabolic activity, performed by MTT (3-(4,5-Dimethylthiazol-2-yl)-2,5-diphenyltetrazolium bromide) (**A**) and Alamar Blue (**B**) assays. Results are reported as mean values and 95.0% least significance difference (LSD) intervals of optical density (**A**) and fluorescence emission (**B**) of hASCs cultured on FAMs after 7 days of culture

Analysis of hASCs metabolic activity (two cell lines) was performed by both MTT (3-(4,5-Dimethylthiazol-2-yl)-2,5-diphenyltetrazolium bromide) and AlamarBlue assays on five batches of FAMs at 3 time points: day 0 (2 h after cell-microcarrier incubation), 1, and 7 days from cell seeding. Mean values and 95.0% least significance difference (LSD) intervals are reported in Figure 8 as optical density (OD, Figure 8A) and fluorescence (Figure 8B) for MTT Assay and Alamar Blue, respectively, during the culture time. Analyses revealed that both OD and fluorescence intensity depend on culture time (*p* values: 0.0002 and 0.0038, respectively). No significant effect was observed between the two cell lines (*p* values: 0.1024 and 0.6042, respectively for OD and fluorescence intensity). Moreover, the batch of FAMs did not influence the hASCs metabolic activity (*p* > 0.05). Results of both metabolic assays clearly suggest that no differences were observed after 2 h of stirring (0 day) and 1 day of culture, but hASCs were able to proliferate on the FAM.

### 2.4. Human ASCs Multi-Differentiative Potential

**Figure 9:**
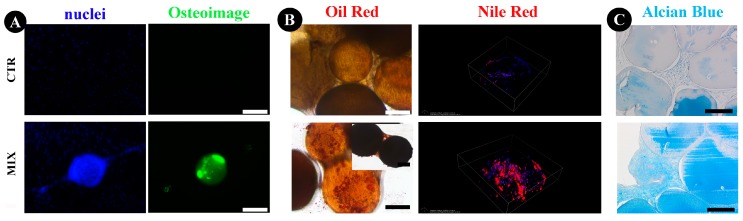
Evaluations of osteogenic, adipogenic, and chondrogenic potential of hASC, cultured on FAMs. Hoechst (nuclei) and Osteoimage stainings for osteogenesis (**A**), Oil Red and Nile Red staining for adipogenesis (**B**), and Alcian Blue staining for chondrogenesis (**C**). Scale bar = 200µm.

Osteogenic lineage differentiation was assessed by analyzing deposition of mineralized matrix and hydroxyapatite (HA) bone-like nodules. On day 28, untreated cells showed no mineralized matrix, while osteogenic-differentiated cells adhered on FAMs were able to produce mineralized matrix (Figure 9A). When cultured in adipogenic conditions, numerous lipid droplets were observed in hASCs cultured on FAMs (Figure 9B). Nile Red staining analysis was performed onto the FAM surface using confocal microscopy selecting only the fluorescence excitation/emission spectra of tryglicerides in order to exclude the Nile Red bound to phospholipids of the bilayer membranes. Figure 9B shows a representative 3D reconstruction of the microcarrier surface where it is possible to distinguish the presence of triglycerides (red) in the treated sample with respect to control.

The histological evaluation after chondrogenic differentiation revealed a higher amount of matrix with higher compactness in the presence of chondrogenic stimulus hASCs with FAMs compared to the control condition (Figure 9C).

## 3. Discussion

Microcarriers represent the ideal cell delivery system in regenerative medicine because of their properties, such as an optimal surface area to volume ratio, promoting cell adhesion and proliferation; microcarriers can also be directly injected in the affected site ensuring cell localization, remaining in the desired target tissue [2], minimizing cell manipulation ex vivo and reducing costs of cell expansion in Good Manufacturing Practice facilities.

In the present work, a new composite microcarrier consisting of an alginate core and a silk fibroin shell was designed as a potential vehicle and delivery system for cells in regenerative medicine purposes. Alginate and fibroin, two natural polymers, were selected because they are biocompatible and widely employed in tissue engineering and regenerative medicine. Alginate was used as a core giving stability to microcarriers, whereas fibroin was added to enhance cell adhesion and proliferation on the biomaterial, as previously reported [30,45]. Recently, we also observed that silk fibroin, manufactured as nanoparticles, was cytocompatible on hASCs and easily internalized by the cells [44].

A recent paper by Lin et al. [46] investigated four commercially available microcarriers (Cytodex 1, Cytodex 3, SphereCol, and Cultispher-S) for improved adhesion and chondrogenic differentiation of human early mesenchymal stromal cells. These microcarriers have different sizes (ranging from 100 to 400 μm), matrices (dextran or collagen), surface nature (positively charged or denatured collagen), and surface appearance (smooth or porous). The authors conclude that matrix material, surface, or even matrix stiffness may affect differentiation outcomes. On the other hand, given that Cytodex 1 (positively charged) and Cytodex 3 (gelatin-coated) achieve similar levels of cell growth and chondrogenic output, they suggest that microcarrier gelatin coating or charges are unlikely to be as crucial in determining differentiation outcomes as the gelatin coating alone did not improve cell growth and differentiation outcomes. 

In agreement with Lin’s conclusions, we propose fibroin as a new protein coating material for alginate microcarriers. Our coating process did not modify the size (about 400 µm) of microcarriers, similar to some of the commercially available cell carriers [47,48]. Coating with fibroin gave a rough surface morphology to microcarriers that could be relevant to increase cell adhesion and thus proliferation. In fact, many authors have already highlighted the importance of mimicking the rough nature of extracellular matrices during scaffold design and production, in order to improve the adhesion and the spreading of cells [49,50]. A silk stable conformation consisting of crystalline β-sheets was observed by FTIR; confocal microscopy investigation showed a homogeneous coating on the alginate surface, with some fluorescent clots related to marked roughness.

Cell-FAM samples analyzed by TEM showed tight contacts between hASCs and fibroin surface while keeping the cell morphology unaltered. The hASCs’ adhesion to microcarrier substrate was also confirmed by immunofluorescent staining, labeling the F-actin with a fluorescent antibody after a cultivation time from 7 days (Figure 6) up to 14 days (data not shown). It is well-known that adhesion is a complex series of specific interactions and recognitions that occur between the extracellular matrix and integrins present on the cell surface. A correct orientation of F-actin filaments determines the spreading-like morphology of the cell body that favors their normal phenotype and functionality. In this study, the hASCs’ adhesion to FAMs was clearly observed through confocal imaging showing dense and well-elongated cell actin fibrils on the microcarrier surface. A few authors correlate these cytoskeletal distributions to the augment of cytoskeletal tension that should improve some cell abilities such as proliferation and differentiation potential [51,52].

FAMs also showed cytocompatibility, since hASCs seeded on FAMs were able to efficiently proliferate. The hASCs had already started to proliferate after 24 h of being seeded onto FAMs and completely covered their surface after 7 days of culture, as reported by Schop et al. for dextran based microcarriers.

Microcarriers were frequently used for MSC expansion and several authors analyzed their proliferation and differentiation capabilities when cultured on these supports. In 2014, Caruso and colleagues used commercial Cytodex 3 microcarriers for MSC expansion and they observed that cells maintained their metabolism as well as their immunophenotypic and functional characteristics [53]. Similarly, MSCs were cultured on fibronectin-coated, non-porous plastic microcarriers in spinner flasks, then detached and cryopreserved. After thawing, cell viability was higher than 70% and cells maintained their adherence ability to plastic surfaces as well as their proliferation [54]. Similar results were published by Yuan and colleagues who demonstrated the maintenance of adipogenic and osteoblastic differentiation of MSCs after static or dynamic cultures on commercial CultiSpher-S microcarriers [55].

In our experiments, hASCs cultured on FAMs maintained their viability and multipotency. Recently, a new approach in regenerative medicine is oriented towards intraoperative solutions based on minimal cell manipulation, such as cell concentrate from bone marrow or adipose tissue [56,57]. In this context, fibroin has been employed as an adipose stromal vascular fraction delivery system to support cell implantation in a murine model [58]. We suppose also that fibroin-coated microcarriers allow for cell adhesion, thus making them suitable for intraoperative use of cell concentrate.

## 4. Materials and Methods

### 4.1. Preparation of Microcarriers

Silk fibroin solution was prepared starting from Bombyx mori cocoons that were degummed in autoclave (Systec V-65, Wettenberg, Germany) to separate fibroin fibers and sericin solution [45,59]. Silk fibroin fibers were rinsed three times with distilled water at 60 °C, dried at room temperature (RT), cut in small pieces and solubilized in phosphoric acid/formic acid (80:20 *v*/*v*) (Sigma-Aldrich, Milan, Italy) while stirring at room temperature. Raw fibroin solution was dialyzed against distilled water using a modified polyethersulfone membrane (cut off 12 kDa, Visking, London, UK) at RT. The final concentration of silk fibroin aqueous solution was 1.5% *w*/*v*. 

Alginate microcarriers (AMs) were prepared from a solution of 1% *w*/*v* sodium alginate medium viscosity (Sigma-Aldrich, Milan, Italy) in distilled water. The solution was added dropwise by a syringe pump through a 0.17-mm diameter nozzle using a bead generator (Encapsulator VAR V1, Nisco Engineering AG, Zurich, Switzerland) with a differential charge of 7 kV into a solution of distilled water containing 100 mM calcium chloride (Sigma-Aldrich, Milan, Italy) with magnetic stirring. When alginate droplets reached calcium chloride solution, calcium ions diffused into the droplets, leading alginate gelation. Alginate microcarriers were collected by filtration and washed twice with distilled water.

For silk fibroin coating, AMs were shaken into silk fibroin solution (volume ratio alginate microcarriers: fibroin solution 1:2) under mild magnetic stirring for 5 min. Microcarriers were collected by filtration and immersed in 96% (*v*/*v*) ethanol (Carlo Erba Reagents, Milan, Italy) for 15 min to induce silk conformational transition and β-sheet formation. The procedure was performed three times to assure homogeneous and complete coating. Fibroin-coated AMs (FAMs) were suspended in ethanol and stored at 4 °C until use.

### 4.2. Characterization of FAMs

#### 4.2.1. Particle Size

Granulometric analysis of microcarriers was performed with a laser light scattering analyzer (Mastersizer 2000, Malvern Instruments Ldt., Worcestershire, UK) equipped with Hydro SM wet sample dispersion unit; the refractive index was set at 1.359 for ethanol. Results are expressed as the mean value of the five replicates for each batch; the volume weighted mean D(4,3) was considered.

#### 4.2.2. Fourier Transform Infrared Spectroscopy (FTIR)

In order to evaluate the fibroin molecular conformation, samples were analyzed before and after fibroin coating, on a Bruker Alpha-E IR Fourier Transform Spectrophotometer (Bruker, Milan, Italy) equipped with a MIRacle attenuated total reflection Diamond crystal cell in reflection mode, using a resolution of 4 cm^−1^. The spectra were collected in the middle IR (400–4000 cm^−1^). 

#### 4.2.3. Scanning Electron Microscopy (SEM)–Energy Dispersive X-ray (EDX) Analysis 

Samples were placed on aluminum stubs and coated with gold/palladium (60%/40% *w*/*w*). The microcarrier morphology, before and after coating, was evaluated by a scanning electron microscope (SEM-FEG) Mira 3 (Tescan, Brno, Czech Republic), mode High Vacuum, 15 kV and secondary electrons (SEs) detector. EDX analysis was carried out with iXRF system and EDS-2004 system (IXRF Systems, Inc. Austin, TX, USA) for revelation.

#### 4.2.4. Confocal Laser Scanning Microscopy (CLSM)

A confocal laser scanning microscopy analysis of fibroin coating was performed using a NikonTiE microscope equipped with a fully automated A1 confocal laser which incorporates the resonant scanner with a resonance frequency of 7.8 kHz allowing high-speed imaging (A1R, Nikon, Amsterdam, the Netherlands). FAMs were washed three times with saline solution (NaCl 0.9%) and placed in 35 mm µ-dish glass bottom (high, Ibidi Gmbh, München, Germany) for imaging acquisition. In order to identify the correct laser source to detect fibroin fluorescence, a sequential excitation of microcarriers was performed at four different wavelengths (405, 488, 561, and 638 nm). The emission signal was separated using a dichroic mirror (500 nm) and the spectral images were acquired in sequential bandwidths of six nm spanning the wavelength range of 500 to 692 nm to generate a lambda stack containing 32 images. The confocal pinhole was set to 4 Airy disk and a 10× Plan-Apochromatic Ph1 condenser annulus and DL 0.25NA objective lens were used.

### 4.3. Cytocompatibility of FAMs, Cell Proliferation and Cell Differentiation Potential

#### 4.3.1. Isolation and Monolayer Culture of Human Stromal Cells (hASCs)

hASCs were isolated from subcutaneous abdominal fat of 3 informed female donors (37 ± 14 years) who underwent aesthetic liposuction. All of the procedures were carried out at Galeazzi Orthopedic Institute (Milan, Italy) with Institutional Review Board approval (M-SPER-014.ver8 for the use of surgical waste, 8 November 2016). Adipose tissues were washed with phosphate buffered saline solution and digested with 0.075% *w*/*v* type I collagenase (Worthington Biochemical Corporation, LakeWood, NJ, USA) at 37 °C for 30 min [60]. Then, the recovered cells were centrifuged at 1200× *g* for 10 min, filtered with a 100 µm cell strainer and seeded in monolayer culture in complete medium (CM), containing Dulbecco’s Modified Eagle’s Medium-HIGH GLUCOSE (DMEM-HIGH, Sigma-Aldrich, Milan, Italy), 10% fetal bovine serum (FBS, HyClone, EuroClone, Milan, Italy), 50 U/mL Penicillin, and 50 mg/mL Streptomycin, 2 mM l-glutamine (Sigma-Aldrich, Milan, Italy) [61,62]. hASCs were then expanded up to 70–80% of confluency. Then, cells were detached using TripLE™ Select Enzyme solution (Life Technologies, Carlsbad, CA, USA) for 3–5 min at 37 °C in 5% humidified CO_2_ atmosphere. Cell pellet, obtained after centrifugation at 450× *g* for 3 min, was mixed with CM and cells were counted using a nucleocounter device (Chemotemec, Allerod, Denmark). hASCs were maintained at 37 °C in a humidified atmosphere with 5% CO_2_, changing culture medium every 3 days; cells up to passage 5 were used for the following experiments. 

#### 4.3.2. Cell Seeding on FAMs

FAMs were washed in saline solution (50% *v*/*v*), conditioned for 24 h in complete medium, and then transferred in 2 mL sterile centrifuge tubes. Afterwards, hASCs were added to the FAM suspension in order to obtain a final seeding density of 5000 cells/cm^2^ of microcarrier surface area [63]. The centrifuge tubes were tightly closed and stirred on an oscillating shaker for 2 h at 37 °C, 5% CO_2_, at 70 rpm to permit the cell adhesion. After the seeding step, fresh medium was added to each tube. 

#### 4.3.3. Cell Viability and Adhesion

The presence of viable cells on FAMs was qualitatively observed with Live and Dead staining (Life Technologies, Monza, Italy) after 1, 3, 7, and 14 days from cell seeding. Briefly, an aliquot of microcarriers was incubated with 2.5 µM of Calcein-AM and 10 µM of Ethidium Homodimer-1 in saline solution for 10 min at 37 °C and 5% CO_2_. After two washes with saline solution, images were acquired using an epifluorescence microscope (Nikon Eclipse TE2000-U inverted epifluorescence microcope) using filter for Calcein-AM: excitation 465–495 nm, emission 515–555 nm; Ethidium Homodimer-1: excitation 510–560 nm, emission 590 nm). 

#### 4.3.4. Metabolic Assays

Five batches of FAMs were tested with hASCs. Metabolic activity of hASCs seeded onto FAMs was measured by the MTT Assay immediately after the stirring period (2 h) and at 1 and 7 days from the cell seeding. A final concentration of 0.5 mg/mL of 3-(4,5-Dimethylthiazol-2-yl)-2,5-diphenyltetrazolium bromide (MTT, Sigma-Aldrich, Milan, Italy) solution was added into each well and after 4 h of incubation at 37 °C and 5% CO_2_, the medium was removed and formazan crystals solubilized in dimethyl sulfoxide. The absorbance of the resulting solution was read at 570 nm (Victor X3, Perkin Elmer microplate, Waltham, MA, USA). 

A further evaluation of the metabolic activity of adherent hASCs was performed by Alamar Blue Assay. In details, aliquots of cell-seeded FAMs were treated with 10% *v*/*v* Alamar Blue solution (Life Technologies) for 4 h at 37 °C. The fluorescence of the obtained solution was measured by Victor X3 (excitation/emission 560/590 nm).

#### 4.3.5. Immunofluorescent Staining for Cytoskeletal Actin 

At each time point (3, 7, and 14 days of culture), hASC-seeded FAMs were fixed with 4% paraformaldehyde solution supplemented with 50 mM CaCl_2_ solution for 45 min with agitation. Samples were washed with saline solution and permeabilized with 0.1% Triton-X (Sigma-Aldrich, Milan, Italy) in saline solution for 15 min. After three washes in saline solution, the cell seeded FAMs were incubated with TRITC-Phalloidin (Sigma-Aldrich, Milan, Italy #P1951, [1:500] in saline solution) for 40 min. The nuclei were stained with 5 µg/mL Hoechst 33342 (Life Technologies, Monza, Italy #H3570, [1:2000] in saline solution) for 10 min. After three washes with saline solution, confocal images were acquired using Nikon A1 confocal laser with a Plan Apo VC DIC N2 20× 0,75NA objective and z-stack setting of 2 µm/step. For TRITC-Phalloidin visualization, a 561 nm laser and a band filter of 570/620 nm range was used.

#### 4.3.6. Transmission Electron Microscopy (TEM)

After 7 and 14 days of culture, samples were fixed with glutaraldehyde 2.5% in 0.1 M cacodylate buffer for 4 h at 4 °C, then with 0.1% osmium tetroxide and finally with alcohols. The scaffold was included in Epon 812/Araldite resin overnight before being sectioned using Ultracut S Ultramicrotome. The thin sections were treated with Toluidine Blue staining (Sigma-Aldrich, Milan, Italy) and observed under an optical microscope while the ultrathin sections were observed for TEM with a JEOL JEM 1200 EX instrument (Jeol, Tokyo, Japan).

#### 4.3.7. Multilineage Differentiation of hASCs

The osteogenic, chondrogenic and adipogenic potentials of hASCs were evaluated, culturing them both in monolayer and on FAMs. 

Osteogenic differentiation was induced by seeding hASCs in 24-well culture plates at 5000 cells/cm^2^; the same density was used to seed cells into 100 µL of microcarriers (50% *v*/*v* of suspension). After a first period of culture in DMEM-HG medium supplemented with 2% FBS, once cells were confluent, the medium was supplemented with 10 mM β-glycerophosphate, 50 µg/mL ascorbic acid, and 1 µM dexamethasone, [64] (all from Sigma-Aldrich, Milan, Italy) and cells were cultured for 28 days changing medium twice a week. Cells cultured in basal medium were used as control. Samples were then stained with OsteoImageTM (Lonza, EuroClone, Pero, Italy) and the assay was carried out according to the manufacturer’s protocol. Briefly, cells were first washed with saline solution and then fixed in 70% ethanol for 20 min at RT. After fixation, samples were rinsed twice with wash buffer and stained with diluted staining solution, while incubating in the dark at RT for 30 min. Finally, samples were rinsed with wash buffer and images were captured using an epifluorescence microscope. 

For adipogenic differentiation, hASCs were seeded at the same density reported above. On the following day, the basal medium was replaced with adipogenic differentiation medium (adipogenic induction medium) composed by DMEM-HIGH, 10% FBS, 1 µM dexamethasone, 0.2 mM indomethacin, 1.75 µM bovine insulin, and 0.5 mM isobutylmethylxanthine (all from Sigma-Aldrich, Milan, Italy). Medium was changed after 3 days and supplemented with 1.75 µM bovine insulin (adipogenic manteinance medium), alternating the two mediums for 21 days [65]. Cells cultured in basal medium were used as control. After 21 days from induction, Oil Red O and Nile Red staining were performed to visualize lipid droplet production. Cultures were washed, fixed with 4% paraformaldehyde in saline solution and stained with 0.3% Oil Red O (Sigma-Aldrich, Milan, Italy) in an isopropanol solution for 15 min with agitation. Finally, the cultures were washed two times with saline solution and images were captured to confirm the lipid droplets. Nile Red staining was done fixing the cultures in 4% paraformaldehyde solution supplemented with 50 mM CaCl_2_ for 10 min at RT, washing three times with saline solution, and finally staining with 5 µg/mL Nile Red for 20 min in dark and RT. Cells’ nuclei were stained with Hoechst as described above. After one wash in saline solution, samples were imaged with confocal with Nikon A1 confocal laser microscope with a Plan Apo VC DIC N2 20× 0,75NA objective and z-stack setting of 2.5 µm/step for a lambda stack containing 86 images for the control and 54 for the induced sample. A 514 nm laser and a band filter of 570/620 nm range was used for Nile Red visualization. 

Chondrogenic differentiation was performed in pellet culture using a StemPro Chondrogenesis Differentiation Kit (Life Technologies, Monza, Italy), whereas DMEM-HIGH supplemented with 10% FBS was used as a control. For pellet culture, 2.5 × 10^5^ cells were mixed with 100 µL of a 50% (*v*/*v*) FAMs suspension in a 15 mL centrifuge tube and spun in a benchtop centrifuge at 450× *g* for 5 min. Pellet cultures without FAMs were used as controls. The samples were put at 37 °C and 5% CO_2_ for one week in a humidified incubator [66]. After one week of incubation, CM (complete medium) was changed in inducing or maintained medium for 21 days (control and induced pellet, respectively), changing the medium twice a week. After differentiation, pellets were fixed for 24 h in 10% calcium formalin, dehydrated and then embedded in paraffin. Four-micron sections were stained with Safranin O to evaluate extracellular matrix deposition. 

### 4.4. Statistical Analysis

The effect of fibroin coating on microcarrier size distribution was assessed by one-way analysis of variance (ANOVA), considering the coating process (Yes or No) as a factor and the volume weighted mean D(4,3) as a dependent variable.

Results on hASCs’ metabolic activity were processed by multifactor ANOVA, considering the time of culture (after 2 h, 1 and 7 days from cell seeding), the cell line (*n* = 2) and the microcarrier batch (*n* = 5) as fixed factors, and the optical density (for MTT assay) and fluorescence intensity (for Alamar Blue assay) as independent variables.

The differences between groups were analyzed with the post-hoc LSD’s test for multiple comparisons. Unless differently specified, data are expressed as mean ± standard deviation. The statistical significance was fixed at *p* ≤ 0.05.

## 5. Conclusions

Our results suggest that the technological process is consistent for the production of fibroin/alginate microcarriers in a lab-scale setting, and the products support hASCs’ adhesion and proliferation. Moreover, hASCs cultured on these 3D systems maintain their ability to differentiate. Therefore, technological resources are available for scale up production: these devices can be used for in vitro expansion/culture as well as for cell transplantation by intraoperative minimally invasive procedure (e.g., injection).

## Abbreviations

AM	Alginate microcarrier
ASC	Adipose mesenchymal Stem Cell
EDX	Energy dispersive X-ray
FAM	Fibroin-coated alginate microcarrier
FTIR	Fourier transform infrared spectroscopy
MSC	Mesenchymal stem cell
